# A Comparative Effect of Nano‐Zirconia and Nano‐Zinc Oxide on Color Stability of Pigmented Maxillofacial Silicone Under Accelerated Aging

**DOI:** 10.1155/ijbm/5375243

**Published:** 2026-08-29

**Authors:** Shahad Mohammed Shakir, Zryan Jaza Hama salih, Shahad Fadhil Bunyan, Izzaldeen Yaseen Aljumaily, Faraedon Mohidden Mostafa Zardawi

**Affiliations:** ^1^ Department of Prosthodontic Technology, Institute of Health and Medical Technology, Middle Technical University, Baghdad, Iraq, mtu.edu.iq; ^2^ Department of Dental Technology, Komar University of Science and Technology, Sulaymaniyah, Kurdistan Region, Iraq, komar.edu.iq; ^3^ Department of Forensic Evidence Techniques, Polytechnic College Al-Hawija, Northern Technical University, Kirkuk 36007, Iraq, ntu.edu.iq; ^4^ Department of Prevention–Orthodontic–Pediatric, College of Dentistry, Kirkuk University, Kirkuk, Iraq, uokirkuk.edu.iq; ^5^ Dean of the Faculty of Dentistry, Qaiwan International University, Sulaimania, Kurdistan Region, Iraq, uniq.edu.iq

**Keywords:** accelerated weathering, color stability, maxillofacial silicone, nano-zinc oxide, nano-zirconia

## Abstract

This in vitro study was designed to assess and compare the color stability of pigmented maxillofacial silicone elastomers reinforced with nano‐zirconia and nano‐zinc oxide with silicone without nanoparticles following accelerated weathering at various time intervals. A total of 150 disc‐shaped pigmented maxillofacial silicone specimens were prepared and classified into one control group of 50 silicone elastomers pigmented without nanoparticles and two experimental groups of 50 nano‐zirconia (ZrO_2_) and 50 nano‐zinc oxide (ZnO). The samples were then exposed to artificial weathering for 200, 400, and 600 h of accelerated weathering. All color measurements were performed at baseline and after aging using same specimens assigned to each interval with a spectrophotometer in the Commission Internationale de l’Eclairage (CIELAB) color space. Color differences (ΔE) were analyzed using two‐way repeated measures ANOVA with Greenhouse–Geisser correction (*p* < 0.05). Color change values of both groups increased markedly with time aging, indicating that nanoparticle reinforcement significantly influenced the color change over time. At all aging intervals, the ZrO_2_ group demonstrated the lowest ΔE values, followed by ZnO, while the control group exhibited the highest discoloration. After 600 h of accelerated aging, mean ΔE values were 3.23 for the control group, 2.82 for ZnO, and 2.16 for ZrO_2_, exhibiting a superior color stability of zirconia‐reinforced silicone. Nano‐ZrO_2_ showed better color change stability than nano‐ZnO at all aging periods, indicating that it may be used to increase the esthetic longevity of maxillofacial silicone prosthesis.

## 1. Introduction

Congenital or acquired facial defects are common problems that often require prosthetic rehabilitation to restore facial form and function, along with psychological normalcy. Silicone elastomers are common in the practice of maxillofacial rehabilitation due to such qualities as flexural strength, biocompatibility, ease of processing, and ability to mimic the texture and appearance of human skin [1]. In spite of these benefits, the long‐term clinical service life of silicone prostheses is generally diminished due to low color stability, which is still desired to be one of the main causes for prosthesis replacement [2].

Color stability is one of the most important esthetic demands for maxillofacial silicone prostheses since slight color change became visible in facial areas [3]. The discoloration of silicone elastomers is a complicated problem involving intrinsic causes such as pigment degradation, filler–matrix interaction, and polymer oxidation as well as extrinsic factors including UV irradiation, humidity variations, temperature changes, and environmental contamination [4]. Exposure to these factors over time can lead to surface degradation, pigment deterioration, and gradual discoloration, causing loss of patient satisfaction [5].

To shorten the experimental time of aging research, maxillofacial materials are typically weathered to simulate years of exposure by using accelerated methods. UV irradiation combined with controlled heat and moisture has shown a good simulation of clinical aging and to duplicate color changes similar to those measured during real service [6]. Therefore, accelerated weathering protocols have been recommended as being valuable tools for the evaluation of the color stability of silicone elastomers and to compare the performance of modified materials under standardized conditions [7].

The nanoparticle reinforcement has been considered as a conducive approach to enhancing physical, mechanical, and optical characteristics of polymer‐based dental materials. Nanoparticles have high surface area and special physicochemical properties, so they can contribute to improve the polymer’s resistance to environmental degradation by preventing the penetration of UV radiation and decreasing oxidative reactions in the polymer matrix [8]. Among different metal oxide nanoparticles, zirconium dioxide (ZrO_2_) has got more attention due to their possible application of enhancing the resistance to aging of maxillofacial silicone elastomers [9].

Nano‐zirconia shows high chemical durability, excellent photostability, and an inactive optical performance, which provides good potentiality for the improvement of color stability in silicone materials [10]. On the contrary, nano‐ZnO possesses excellent UV‐absorbing ability and antimicrobial property. However, it is reported that ZnO nanoparticles have some responsibility on yellowing after long‐time aging [11, 12].

Consequently, an in vitro study comparing these particles under the same experimental condition is essential to assess their comparative efficacy toward the color stability. Although the mechanical reinforcement of maxillofacial silicone elastomers with these nanoparticles has been reported in previous studies, an insufficient amount of data exists regarding their comparative effects on color stability during prolonged aging periods [4]. In addition, the appearance of progressive color development over time has only been evaluated using accelerated weathering protocols at multiple time points in a small number of previous studies. Hence, this study was designed to assess and compare the color stability of pigmented silicone reinforced with nano‐zirconia and nano‐zinc oxide with pigmented without nanoparticle silicone elastomers before and after 200, 400, and 600 h of accelerated weathering. The null hypothesis was that the two nanoreinforced groups would exhibit similar color stability over the various aging durations.

## 2. Methodology

### 2.1. Study Design and Grouping

This in vitro experimental study evaluated the color stability of pigmented maxillofacial silicone elastomers reinforced with nano‐zirconia and nano‐zinc oxide following accelerated weathering. Color changes were assessed at three aging intervals (200 h, 400 h, and 600 h) compared to pigmented silicone without nanoparticles using spectrophotometric analysis based on the Commission Internationale de l’Eclairage (CIELAB) color system.

Specimens were randomly divided into three groups: one control group of 50 pigmented silicone without nanoparticles and two experimental groups based on the type of nanoparticle reinforcement, namely, 50 pigmented silicone reinforced with nano‐zirconia (ZrO_2_) and 50 pigmented silicone reinforced with nano‐zinc oxide (ZnO). G ∗ Power (3.1.9.7) was used to perform an a priori power analysis to identify the minimum required sample size of an ANOVA with repeated measurements and three measurement time points with three groups. With a medium effect size (*f* = 0.25), alpha = 0.05, and a statistical power of 80, the total size of the sample was found to be 108 specimens (36 specimens per group). In order to improve statistical accuracy and counter the possible variability of the experiment, 50 specimens were assigned per group that would give an overall sample size of 150 specimens.

### 2.2. Materials

A commercially available medical‐grade maxillofacial silicone elastomer Tech‐Sil25 (Technovent Ltd., Bridgend, UK) room temperature vulcanized with two parts, the base, and catalyst were combined in a 9:1 ratio according to manufacturer specifications with yellow (Technovent Ltd., Bridgend, UK) pigments to simulate clinically relevant facial prosthetic coloration and to allow detection of yellowing shifts (b∗ parameter) during aging. The research uses nano‐zirconia (ZrO_2_) particles (7631‐86‐9), characterized by 99.9% purity and a particle size of 30–50 nm, and nano‐zinc oxide (ZnO) (1330‐060920), distinguished by 99.9% purity and a particle diameter of 40–60 nm, as the nanofiller materials.

### 2.3. Specimen Preparation

**FIGURE 1:**
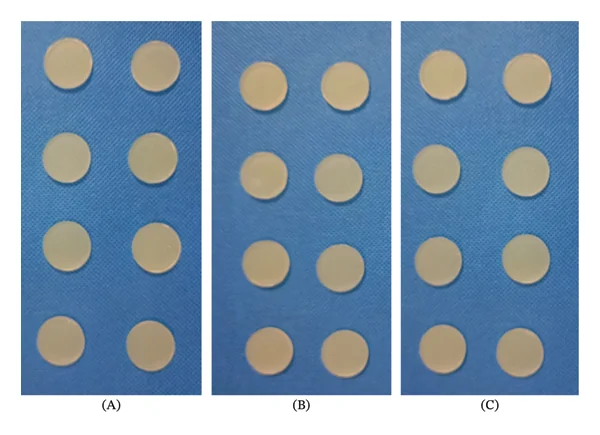
(A) Nano‐zirconia (ZrO_2_) group, (B) nano‐zinc oxide (ZnO) group, and (C) control group.

Disc‐shaped specimens were fabricated using custom molds fabricated using a Computer Numerical Control (CNC) milling machine with dimensions of 2‐mm thickness and 20‐mm diameter specimens, as shown in Figure 1. The vacuum mixer was used to make the specimens. It blended Part A, Part B, and the nanomaterial with 0.2 wt% dry earth pigment. To make sure the nanoparticles mixed well with the silicone foundation and got rid of any extra nanoparticles, they were first placed into the mixing container with a clean spatula and weighed with a digital electronic weight balance (Iscale, China). A mechanical mixer (Degosa, Germany) combined the silicone base with nanoparticles at 360 rpm and −10 bar pressure. The base and nanomaterial were mixed together for 10 min before the colorant was added and blended for 5 min with a vacuum mixture. After that, Part B silicone was weighed and added to the mixing bowl. The mixture was mixed for 5 min and 5 s using a vacuum mechanical mixer [10]. To keep the temperature and humidity at the right levels, we used a digital thermometer and hygrometer sensor to keep an eye on the standard condition. The temperature should be between 23°C and 2°C, and the humidity should be between 50% and 10%.

When pouring the samples, air bubbles will form and rise to the top, which were removed with a very tiny stainless‐steel dental probe before covering. The load was taken off after the screws were tightened using nuts and G‐clamps. For a consistent result, the silicone samples were kept in a lightproof box in an air‐conditioned room. The temperature stayed between 20°C and 25°C, and the humidity stayed below 60% for 16 h. The samples were stored in the dark from the time they were vulcanized until they were tested. According to ASTM D624 (2012), the minimum duration for vulcanization is 16 h, stored at normal humidity of 50 ± 10% to prevent them from becoming wet [12]. The molds were filled with the mixture and closed and pressure was applied. The samples were allowed for 24 h to finish the vulcanization process. After that, the samples were taken out of the molds and put through the weathering process [13].

### 2.4. Accelerated Weathering Protocol

The UV aging chamber was employed to implement the accelerated weathering for simulating long‐term environmental exposure. Specimens were exposed and cured under continuous UV irradiation in a controlled temperature and humidity environment. The aging was then performed at 200, 400, and 600 h, corresponding to increasing clinical service times. UV aging has been well accepted as an effective means of simulating the long‐term color degradation of maxillofacial silicone materials in a relative short period [14, 15].

### 2.5. Color Measurement

Color measurements were performed using a calibrated spectrophotometer operating under standardized illumination conditions. Measurements were recorded at baseline before aging and after each aging interval. The CIELAB color coordinates (*L* ^∗^, *a* ^∗^, and *b* ^∗^) were obtained for each specimen, and color differences (ΔE) were calculated using the following standard formula:

(1) ΔE=ΔL∗2+Δa∗2+Δb∗2.

The CIELAB system is widely used for evaluating color changes in dental and maxillofacial materials due to its sensitivity and clinical relevance [16].

### 2.6. Statistical Analysis

Analysis of data was done using SPSS (IBM SPSS Statistics, Version 28, IBM Corp., Armonk, NY, USA). A two‐way repeated measures ANOVA was conducted to determine the effects of the age of time within‐subject factor and nanoparticle group between‐subject factor on the ΔE values. Sphericity was evaluated using the Mauchly test, and Greenhouse–Geisser was used where the assumption failed. The statistical significance level was established at *p* under 0.05 [17].

## 3. Results

**FIGURE 2:**
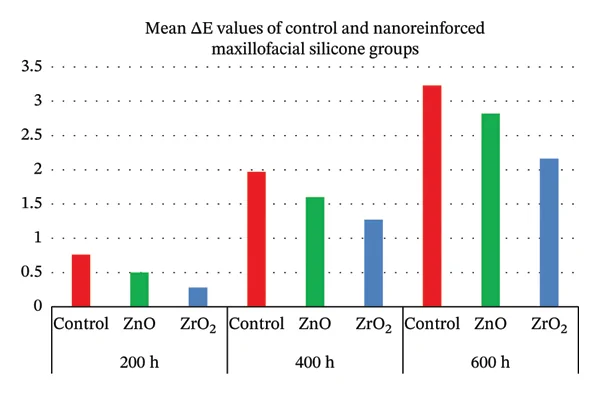
The progressive increase in ΔE values for both nanoparticle groups across the three aging intervals.

Table 1 and Figure 2 show the descriptive statistics of the studied groups’ color change (ΔE) values across aging intervals. After 200 h, the control group exhibited the highest mean ΔE value (0.76 ± 0.09), followed by the ZnO group (0.50 ± 0.06), while the ZrO_2_ group demonstrated the lowest color change (0.28 ± 0.05). After 400 h, ΔE values increased markedly in all groups. The control group showed a mean value of 1.97 ± 0.11, ZnO recorded 1.60 ± 0.15, and ZrO_2_ showed 1.27 ± 0.09. At 600 h, the highest degree of discoloration was observed. The control group showed a mean ΔE of 3.23 ± 0.06. ZnO showed a value (2.82 ± 0.09), while ZrO_2_ was the lowest ΔE (2.16 ± 0.09).

**TABLE 1:** Descriptive statistics for the studied group.

Aging time: group		*N*	Mean	Std. deviation	Std. error	95% confidence interval for mean		Minimum	Maximum
						Lower bound	Upper bound		
200 h	Control	50	0.76	0.09	0.012	0.73	0.7894	0.56	0.92
	ZnO	50	0.50	0.06	0.008	0.48	0.5194	0.31	0.65
	ZrO_2_	50	0.28	0.05	0.007	0.26	0.2988	0.11	0.36

400 h	Control	50	1.97	0.11	0.016	1.93	2.0040	1.60	2.30
	ZnO	50	1.60	0.15	0.021	1.55	1.6438	1.29	1.91
	ZrO_2_	50	1.27	0.09	0.013	1.25	1.3035	1.11	1.49

600 h	Control	50	3.23	0.06	0.009	3.21	3.2551	3.11	3.37
	ZnO	50	2.82	0.09	0.012	2.80	2.8549	2.60	2.99
	ZrO_2_	50	2.16	0.09	0.013	2.14	2.1960	2.01	2.39

Mauchly’s test indicated that the assumption of sphericity was violated (*W* = 0.861, *χ* ^2^ (2) = 21.880, *p* < 0.001). Therefore, Greenhouse–Geisser correction (*ε* = 0.878) was applied. Table 2 shows that a two‐way repeated measures ANOVA with Greenhouse–Geisser correction revealed a significant main effect of time (*F* = 21137.098, *p* < 0.001), indicating a progressive increase in ΔE values across aging intervals. A significant time × group interaction was also observed (*F* = 138.938, *p* < 0.001), confirming that nanoparticle reinforcement significantly influenced overall color stability. Post hoc comparisons revealed that the ZrO_2_ group exhibited significantly lower ΔE values.

**TABLE 2:** Tests of within‐subjects effects using the Greenhouse–Geisser correction.

Source		Type III sum of squares	Df	Mean square	*F*	Sig.
Time	Greenhouse–Geisser	372.342	1.756	212.081	21137.098	< 0.001
Time ∗ Group	Greenhouse–Geisser	4.895	3.511	1.394	138.938	< 0.001

## 4. Discussion

The present results showed that nanoparticle incorporation significantly influenced the color stability of maxillofacial silicone elastomers during accelerated aging. Both nanoparticle‐reinforced groups exhibited lower color change values than the control group throughout the study period. Among the tested materials, nano‐zirconia showed the greatest resistance to discoloration, while nano‐zinc oxide exhibited intermediate performance. Therefore, the null hypothesis was rejected.

The gradual increase in ΔE at 200, 400, and 600 h of accelerated weathering is supported by previous studies which showed that the ultraviolet aging–induced degradation in maxillofacial silicone elastomers was time dependent [18]. Ultraviolet radiation is the major cause for induction of photo‐oxidative reactions in the silicone matrix that leads to polymer chain scission, surface roughening, and pigment instability, causing discoloration over an observed period of time [19].

Nanofiller‐containing composites disclosed the best color stability at all aging times. The relatively bioinert and UV‐protective capacity of zirconia nanoparticles is perhaps contributing to this result. Zirconia NPs can act as physical filters, hindering UV light infiltration into the silicon network and modulating pigment decomposition and polymer oxidation [20, 21]. The slightly lower ΔE values observed in the nano‐zirconia group remained below clinically unacceptable thresholds even after 600 h of aging, indicating improved long‐term esthetic stability.

The nano‐zinc oxide–filled samples, however, exhibited much higher ΔE values especially after extended aging. UV‐absorbing properties of zinc oxide nanoparticles contribute to an enhanced photocatalytic activity under UV illumination, which in turn may result in oxidative reactions within the polymer matrix, causing more yellowing and color instability [4, 12]. This is manifested in higher values for the nano‐zinc oxide group, demonstrating enhanced yellowing after 600 h of weathering.

The CIELAB coordinate analysis also confirmed these observations as ΔL values of negative value for both groups, reflecting a darkening after aging, were found, which is in agreement with literature on silicone elastomer degradation [22, 23]. Nevertheless, the more marked color changes of nano‐zinc oxide samples indicate that yellowing is a viable discoloration mechanism in ZnO‐modified silicone probably affecting its esthetic stability in facial prostheses [24].

The present study tested color stability by the conventional CIELAB color difference equation (ΔEab), which has been widely employed in dental and maxillofacial materials studies due to its simplicity and usefulness in quantifying the overall color difference between two color measurements in the CIELAB color coordinates (*L*, *a* ^∗^, and *b* ^∗^). The ΔE∗ab formula assumes, however, that the CIELAB color space is perceptually uniform, that is, a numerically equal difference is visually equal on all colors. This assumption has been shown to be incorrect by subsequent research because human color perception is a function of lightness, chroma, and hue. For this reason, the CIE introduced the CIEDE2000 (ΔE00) equation, which is a more clinically relevant color difference metric by providing greater agreement with human visual perception of color differences between instrumental measurements. The CIEDE2000 equation differs from the conventional ΔEab equation by incorporating mathematical correction terms for lightness, chroma, and hue differences and perceptual weighting functions as well as an interaction term that accounts for the nonuniformity of the CIELAB color space. Therefore, generally, ΔE00 should be taken as a more reliable indicator of the probability of an observer detecting or finding a color difference clinically acceptable [25].

Clinically, keeping the ΔE limits lower than what is perceptible and acceptable is important for patient satisfaction and preservation of prosthesis. The present investigation showed that nano‐zirconia containing maxillofacial silicone keeps color stability better than the nano‐zinc oxide over a long period of aging compared to the control group. This result is, therefore, important for facial prostheses since even small differences in color are detectable.

However, there were some limitations in this study that should be noted. As an in vitro study, the accelerated weathering test may not entirely simulate the diverse clinical environment, e.g., skin secretions, cosmetics, and mechanical cleaning that would be met. It was only one concentration of nanoparticles tested. Subsequent experiments will focus on varying the concentration and mixing ratio of nanoparticles, as well as longer aging times, in order to further optimize color stability and clinical effectiveness. An additional drawback of the present study was the use of the conventional CIELAB (ΔE∗) instead of the CIEDE2000 (ΔE00) equation to determine color differences. The CIELAB method is a widely reported method for analysis of maxillofacial silicone materials and is useful for comparing previous publications, but the CIEDE2000 formula is now deemed to be more perceptually accurate due to its greater ability to represent nonuniformities of human color perception. Thus, color differences reported may not completely represent clinically perceived color changes. Future studies are encouraged to use the CIEDE2000 calculations to offer a more clinically relevant measurement of color stability.

## 5. Conclusion

Silicone samples reinforced with nano‐zirconia exhibited color stability that was significantly higher than upon reinforcement with nano‐zinc oxide at all aging times compared to the control group, which showed ΔE at clinically acceptable limits even after long‐term exposure to accelerated weathering, while the nano‐zinc oxide specimens demonstrated more significant color change and additional yellowing with longer exposures; the addition of nano‐zirconia to be present in maxillofacial silicone materials may help to enhance esthetic durability and service life with regard to a facial prosthesis. Additional studies with varying nanoparticle concentrations, combination nanoparticles, and long‐term clinical assessments can be performed to optimize material performance and confirm the validity of these results in a large clinical setting.

## Funding

The research was self‐funded.

## Conflicts of Interest

The authors declare no conflicts of interest.

## Data Availability

Data used to support the findings of this study are available on request from the corresponding author.
